# Aging effect on the instrumented Timed-Up-and-Go test variables in nursing home women aged 80–93 years

**DOI:** 10.1007/s10522-017-9717-5

**Published:** 2017-06-20

**Authors:** Ryszard Zarzeczny, Agnieszka Nawrat-Szołtysik, Anna Polak, Jakub Maliszewski, Adam Kiełtyka, Beata Matyja, Magdalena Dudek, Joanna Zborowska, Adam Wajdman

**Affiliations:** 1 Institute of Physical Education, Tourism and Physiotherapy, Jan Długosz University in Częstochowa, 13/15 Armii Krajowej Str., 42-200 Czestochowa, Poland; 2 The Jerzy Kukuczka Academy of Physical Education in Katowice, 72A Mikołowska Str., 40-065 Katowice, Poland; 3 TECHNOMEX - Trade and Service Company, 15 Szparagowa Str., 44-100 Gliwice, Poland; 4 BetaMed Medical Center, 100A/802 Mikołowska Str., 40-065 Katowice, Poland; 5 Saint Elisabeth Nursing Home in Ruda Śląska, 30 Wolności Str., 41-700 Ruda Slaska, Poland

**Keywords:** Aged, Inertial unit, Functional capacity, Acceleration, Knee extension, Muscle strength

## Abstract

Although the total “Timed-Up-and Go” test (TUG) performance time can characterize an age-related decline of general mobility, this result alone doesn’t give any detailed information about the test subtasks. The primary objective of the study was to identify in nursing home women a variable extracted from instrumented TUG (iTUG) that is the best predictor of age. The secondary objective was to assess whether this variable is associated with the results of the isometric knee extension peak torque (IKEPT); lower limb strength measured by the 30-s chair stand test (30sCST), and walking capacity measured by the 6-min walk test (6MWT). Twenty-six women (mean ± SD: age—85.8 ± 3.6 years; body weight—59.4 ± 12.3 kg; body height—151.0 ± 7.3 cm; BMI—26.0 ± 4.9 kg/m^2^) performed iTUG (while wearing a body-fixed inertial sensor) and functional tests. Total iTUG performance time significantly correlated with age (r = 0.484; p < 0.05), 30sCST (r = −0.593; p < 0.01), and 6MWT (r = −0.747; p < 0.001) but not with absolute nor relative IKEPT (p > 0.05). Additionally, the subjects’ age correlated with 30sCST (r = −0.422; p < 0.05), 6MWT (r = −0.482; p < 0.05), IKEPT (r = −0.392; p < 0.05) and IKEPT/FFM (r = −0.407; p < 0.05). Five out of 16 analyzed iTUG variables were significantly related to age, and multiple regression analysis showed the best correlation with the sit-to-stand vertical acceleration range (STSVAR) (r^2^ = 0.430; SEE = 3.041; β = −0.544 ± 0.245; B = −1.204 ± 0.543; p < 0.05). Moreover, STSVAR was significantly associated with %Fat (r = 0.415; p < 0.05), 30sCST (r = 0.519; p < 0.01), 6MWT (r = 0.585; p < 0.01) but not with absolute nor relative IKEPT (p > 0.05). The obtained results suggest that in the oldest old group of nursing home women an age-related decline in TUG performance is mainly associated with a reduction of “explosive” strength of lower limb muscles.

## Introduction

In elderly people, the important component of the so called “health related quality of life” is a functional status i.e. the capacity to perform basic activities of daily living (ADL). Among these activities mobility seems to play the central role (Bentley et al. 2013). Tasks such as climbing stairs, getting out of a chair or bath tub, walking across a room, rising from a horizontal position and maintaining standing balance are predictive of disability, institutionalization, and mortality (Guralnik et al. 1995). In fact, limitations in mobility affects 25% of individuals aged 65 years and older, and the prevalence of these limitations in nursing home residents is even higher reaching the value of 75% (Sackley et al. 2009). These numbers indicate that special attention in research programs should be given to the prevention of functional decline in the institutionalized older people.

It is well established that mobility limitations in elderly individuals are associated with an age-related decline in muscle properties and function (Goodpaster et al. 2006). However, there is no a specific age at which mobility drawbacks occur since the muscle weakness could be significantly different even in the persons at the same age. Two terms have been introduced into scientific literature to identify the threshold in the aging process: sarcopenia and frailty. According to The European Working Group on Sarcopenia in Older People (EWGSOP) sarcopenia is “a syndrome characterised by progressive and generalised loss of skeletal muscle mass and strength with a risk of adverse outcomes such as physical disability, poor quality of life and death” (Cruz-Jentoft et al. 2010). On the other hand, frailty has been defined as a geriatric syndrome, directly associated with age, characterized by a multisystem reduction in physiological capacity leading to increased vulnerability for adverse health outcomes (Chen et al. 2014). Although these two health conditions are related to one another, sarcopenia is thought to be the final common pathway in the pathogenesis of frailty (Morley et al. 2014). Taking into account that frailty as well as sarcopenia, differently from disability, are described as reversible conditions (Michel et al. 2015; Montero-Fernández and Serra-Rexach 2013), there exists the need for substantial investigation on the age-related factors which can potentially contribute to frailty and/or sarcopenia in order to conceive screening tools to identify persons at risk of functional decline.

Surveys on functional status in older people frequently include performance-based tests to stratify the risk of muscle weakness. These tests focusing mainly on walking ability, muscle strength and balance, often assess only one physical variable (e.g. time, distance, number of repetitions) and do not provide any information on the specific aspects of the test subtasks. A typical example of such tests is Timed-Up-and-Go test (TUG)—a simple, quick and widely used measure of functional mobility. The time taken to complete TUG has been shown to be linked to regular physical activity (Riebe et al. 2009), global health decline (Viccaro et al. 2011), disability in activities of daily living (Wennie Huang et al. 2010), and falls (Beauchet et al. 2011). Recently it has been reported that TUG performance time can be a strong predictor of frailty (Savva et al. 2013) as well as sarcopenia (Martinez et al. 2015). However, keeping in mind that both of these health conditions are related to muscle weakness, the TUG performance time alone does not provide any detailed information concerning the test components i.e. (sit-to-stand and stand-to-sit transitions, straight-line walking and turning) that might be particularly sensitive as predictors of functional decline.

Advances in technology made possible to perform a measurement of acceleration and angular velocity in 3 directions. Further, this fact has led to the development of small and lightweight devices with inertial sensors which can be attached to the body (Sprint et al. 2015). Using such equipment, it has been reported that the variables extracted from the instrumented TUG (iTUG) are more accurate than the total TUG duration at distinguishing frail from non-frail community-dwelling elderly (Greene et al. 2014) and young from older adults (Vervoort et al. 2016). To the best of our knowledge, no studies have examined a relationship between age and iTUG variables in the oldest old group of people living in residential care units.

In view of the presented findings and paucity in the literature, our primary objective of this study was to identify an iTUG variable that is the best predictor of age in a nursing home residents sample of women aged 80–93 years. The secondary objective was to assess whether this variable is associated with the results of the 30-s chair stand test (30sCST), the 6-min walk test (6MWT) and the isometric knee extension peak torque (IKEPT).

## Materials and methods

### Participants and study design

**Fig. 1:**
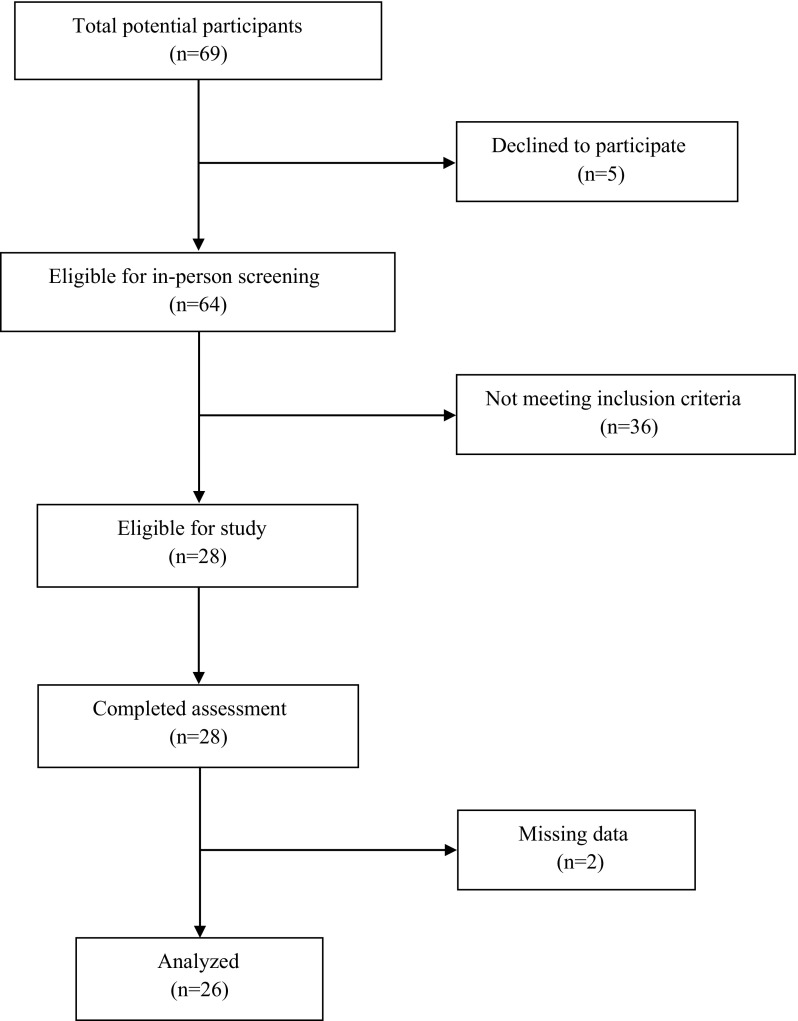
Flow chart displaying of recruitment process of investigated elderly women

In this cross-sectional analysis, a sample of twenty-six women, 80 years of age or older were recruited from the Upper Silesia nursing homes in Poland. Participants were screened through a medical history questionnaire and physical examination. The inclusion criteria were: the age 80 years old and older, ability to perform independently basic activities of daily living [scoring 6 points according to Katz Index of Independence in Activities of Daily Living (Wallace and Shelkey 2008)], preserved logical verbal contact, lack of medical contraindications to physical exercise. Subjects ability to perform basic ADL (i.e. bathing, dressing, toileting, transferring, continence and feeding) was assessed by a home care nurse. Medical contraindications to physical exercise was assessed by a geriatric physician on the base of subjects medical history, physical examination, and ability to walk 20 m without assistance or resting (Donat Tuna et al. 2009). Subjects were excluded if they had a cancer, uncontrolled high blood pressure or used diuretics, had an atrial fibrillation, implanted cardiac pacemaker, amputations, epilepsy, neurodegenerative disorders, used walking aids, currently experienced pain or aching in joints or experienced them on most days for at least one month during the prior year. The flow of the participants through the trial is shown in Fig. 1.

All women were subjected to anthropometric measurement, body composition analysis, IKEPT measurement and to the standard functional tests such as the iTUG, 30sCST and 6MWT. The anthropometric evaluation, body composition analysis as well as IKEPT measurement were accomplished at the beginning of the study within the same day. The iTUG, 30sCST and 6MWT were performed in random order; however, each functional test was performed only once, and only one test was performed per day. An interval between the tests was 2–3 days. Prior IKEPT, 30sCST and iTUG participants warmed-up by performing a long-distance corridor walk and stretching exercises. There was no warm-up before 6MWT. Data collection was conducted by six experienced assessors (physical therapists) and handling of technical equipment was provided by a qualified engineer. Testing and measuring were carried out at similar times in the morning (9.00–11.00), in similar environmental conditions (20–22 °C at 40–50% humidity), and except of the anthropometric evaluation and body composition analysis (fasting state), 2 h after a light meal. The participants were instructed not to engage in strenuous activity the day before the exercise tests, and for 3 days before and during the study to consume all served in nursing home drinks and liquid meals (c.a. 1500 ml of water). The purpose and risks of the study were explained to each participant before the examination, and written informed consent was obtained from all participants. The study design conformed to internationally accepted policy statements regarding the use of human subjects and was approved by the Bioethics Committee at the Jan Długosz University in Częstochowa.

### Anthropometric and body composition analysis

Anthropometric measurements included body weight and body height. Height was measured to the nearest 0.5 cm using a fixed stadiometer, while the subjects wore light clothing and no shoes. Body weight was measured to 0.1 kg using a body composition analyzer. Body mass index (BMI) was calculated as weight (kilogram) divided by the square of height (meter). Body composition was estimated with a single-frequency bioimpedance analyzer Tanita BC 420MA (Japan). After entering into the BIA machine subjects’ height, age and sex, the subject (in bare feet and light clothing) was instructed to stand with her legs straight, feet parallel with the heel and forefoot placed on the metal plates of the leg-to-leg BIA system. A subthreshold electrical constant current (50 kHz, 90 μA) was then transmitted through the body. On the basis of bioelectrical impedance, fat free mass (FFM) and percentage of body fat (%Fat) were determined using the manufacturer’s in-built equations derived from DXA method (Body Composition Analyzer BC-420MA).

### Isometric strength testing

Given that a mobility decline may be mediated by decreases in muscle strength (Silanpää et al. 2014), and that quadriceps muscle strength is critically important to enable functionally impaired elderly to arise from a chair (Hughes et al. 1996), a measure of a knee extension peak isometric torque was chosen for this study. It has been shown, that this kind of measurement involves a comfortable posture and movement for the subjects, and is readily accepted even by frail individuals (Ploutz-Snyder et al. 2002). Isometric torque of the dominant leg [determined by the Ball Kicking test (Springer et al. 2007)] was measured using a dynamometer system in isometric mode (Jupiter, AC International East, Poland). The subjects sat on knee extension chair in an upright position, with the knee and hip joints at 90° of flexion and the lever arm of the extensor chair was placed on the ankle above the malleoli (Silanpää et al. 2014). The women were asked to exert maximal isometric force over a 2- to 3-s time period. Strong verbal encouragement was used during the test. The torque generated was displayed on the screen of a PC monitor to provide visual feedback. Three test trials were performed, with a 90 s rest between the efforts. The highest value of knee extension torque was used for analysis (Silanpää et al. 2014).

### Functional tests

The main interest of this study was the Timed-Up-and-Go test (TUG) as a widely used clinical performance-based measure of lower limbs function, mobility and fall risk (Herman et al. 2011). Given that TUG incorporates multiple activity themes (sit-to-stand and stand-to sit transitions, walking and turnings) we chose to this study two additional tests: the 30-s chair stand test (30sCST) and the 6-min walk test (6MWT) which mimic TUG components. The 30sCST has been selected since it assesses lower body strength during sit-to-stand and stand-to-sit transitions (Jones et al. 1999). The 6MWT is a commonly used exercise test in clinical practice and research. The 6MWT is considered to provide a valid and reliable measure of functional exercise capacity in patients with cardiovascular and pulmonary disease but also in healthy elders since 6MWT intensity mirrors the efforts frequently performed by elders during normal daily activities (Steffen et al. 2002; Bautmans et al. 2004). It has been reported that in healthy older adults 6MWT results are correlated with knee extension strength and power, habitual and maximal gait velocity, tandem balance as well as with the results of TUG, CST or Berg balance test (Bean et al. 2002b; Harada et al. 1999; Caballer et al. 2015). Taking also into account that in the 6MWT there are changing directions during walking (turnings), what may be especially important for TUG performance in individuals with instrumental ADL disability (Weiss et al. 2013), all these facts contributed to our decision to select the 6MWT into this study.

In order to assess the subjects’ functional ability the following tests were performed:

- *instrumented Timed*-*Up*-*and*-*Go test (iTUG)* while wearing a body-fixed sensor-unit participants were asked to rise from a chair, walk as fast as was safely possible to a line on the floor three meters away, make a 180° turn and walk back to the chair and sit down again (Greene et al. 2014). Shorter total i-TUG times were considered as of better performance. No physical assistance was given during the test. The chair had no armrests and the seat was 46 cm high. The subjects walked through the test once before being timed to become familiar with the test. During testing participants wore an wireless inertial sensing unit (70 × 40 × 18 mm; 37 g; G-Sensor^®^, BTS Bioengineering S.p.A., Italy) fixed with an elastic belt at the level of lumbar segment L5 over the subject’s clothes. The unit consisted of a tri-axial accelerometer and tri-axial gyroscope sensors (1000 Hz sample frequency). The signals were transmitted to a computer via Bluetooth. The iTUG sub-phases times, trunk accelerations and angular velocities were determined using the manufacturer’s software (BTS G-Walk^®^);
- *the 30*-*s chair stand test (30sCST)* participants were asked to stand up and sit down from a chair as many times as possible within 30 s. Arms were crossed over the chest. The chair had no armrests and the seat was 46 cm high. The participants were instructed to sit on the chair without touching the backrest with feet approximately shoulder-width apart placed on the floor at an angle slightly back from the knees, and stand fully during each repetition. The higher total number of stands executed within 30 s was considered as of higher participant’s lower body strength (Millor et al. 2013);
- *the 6*-*min walk test (6MWT)* participants were asked to cover as much distance as possible within 6 min, but were allowed to slow down or stop as necessary (Enright et al. 2003). Standardized verbal instructions were given at the end of each minute to inform of progress and the amount of time remaining. The distance (m) covered in the 6-min walk was recorded and used in the analysis. All safety issues recommended by American Thoracic Society statement (2002) regarding this test were maintained during the measurement. The test was performed using a 30 m walking course in the internal hallway (next to the intensified supervision, examination room), and the distance was marked every 3 m. Each participant was tested individually, and the 6MWT score was assessed by a physical therapist. Throughout the measurement, a registered nurse monitored signs and symptoms for termination of the test. Before and after the 6MWT, heart rate and the modified Borg scale rating were determined. The distance (m) covered in the 6-min walk was recorded and used in the analysis. The longer distance covered within 6 min was considered as of better functional exercise capacity.

### Statistical analysis

The distribution of the data was checked for normality using a Shapiro–Wilk test. The Kruskal–Wallis test was used to detect statistically significance difference between the subgroups of participants with iTUG total time of 10.01–20.00 s; 20.01–30.00 s and >30.01 s. The Mann–Whitney U test compared iTUG measures between the subgroups of ≤13.5 s and >13.5 s of total iTUG time. Variables not normally distributed were log-transformed before correlation analysis. Pearson’s product moment correlation coefficient was used to determine the associations between subjects’ age and iTUG variables. Standard multiple regression analysis was performed to identify the iTUG variable which is the best related to the subjects age. Only those variables that resulted in a statistically significant correlations with subjects’ age were included. The calculations were made using a commercial, statistical software STATISTICA 7.0 (Statsoft, Poland). The level of p < 0.05 was considered significant. The data are presented as means ± standard deviations (±SD) for normally distributed variables and as medians (IQRs-interquartile ranges) for non-normally distributed variables.

## Results

Twenty-six women with a mean age of 85.8 ± 3.6 years were studied. The sample consisted of 4 participants aged 80–82 (15.4% of all investigated women), 8 participants aged 83–85 (30.8%), 9 participants aged 86–88 (34.6%), and 5 participants aged 89–93 (19.2%). Descriptive statistics of the subjects are presented in Table 1.

**Table 1:** Participants characteristics (n = 26)

Variable	Mean ± SD or median (IQR)	Range
Age (years)	85.8 ± 3.6	80.0–93.0
Body height (cm)	151.0 ± 7.3	130.0–162.0
Body weight (kg)	59.4 ± 12.3	38.5–87.7
BMI (kg/m^2^)	26.0 ± 4.9	17.5–36.3
Body fat (%)	30.2 ± 10.4	10.1–46.3
FFM (kg)	40.6 ± 5.9	32.0–54.4
iTUG total performance time (s)	21.06 (9.25)	11.24–50.26
IKEPT (Nm)	21.7 (32.3)	6.0–71.4
IKEPT/body weight (Nm/kg)	0.42 (0.42)	0.12–1.61
IKEPT/FFM (Nm/kg)	0.59 (0.68)	0.16–1.92
30-s CST (repetitions)	7.9 ± 2.7	3.5–15.0
6MWT (m)	149.6 ± 68.5	51.0–300.0
Average gait velocity (m/s)^*^	0.42 ± 0.19	0.14–0.83

* Based on 6MWT results

The iTUG total performance time varied widely between the women and ranged from 11.44 to 50.26 s. Nevertheless, it was significantly correlated (r = 0.484; p < 0.05) with the subjects’ age. Moreover, iTUG total performance time was significantly, negatively associated with the results of 30 sCST (r = −0.593; p < 0.01) and 6MWT (r = −0.747; p < 0.001) but not with the absolute (r = −0.134, p > 0.05) nor the relative values of IKEPT (IKEPT/body weight—r = −0.060, p > 0.05; IKEPT/FFM—r = −0.119, p > 0.05).

Data stratification of iTUG total performance time revealed that the majority of investigated subjects (n = 21; 80.8%) performed iTUG in less time than 30 s, and only 3 women (11.5%) performed this test in less time than 13.5 s (Table 2).

**Table 2:** Participants stratification by their total iTUG performance time (n = 26)

ITUG analysis duration (s)	Subjects number (%)	Mean age ± SD	Age range
0.00–10.00	0	–	–
10.01–20.00	11 (42.3%)	84.0 ± 3.6	80–92
20.01–30.00	10 (38.5%)	86.9 ± 3.3	81–93
>30.01	5 (19.2%)	87.4 ± 2.9	83–91
<13.5	3 (11.5%)	81.7 ± 2.9	80–85
≥13.5	23 (88.5%)	86.3 ± 3.4	80–93

Sixteen variables extracted from iTUG were taken to the analysis (data for the two walking phases—forward and backward gait—were combined). For the whole sample of women, only 5 of iTUG variables namely: sit-to-stand phase duration (p < 0.05), sit-to-stand vertical acceleration range (p < 0.01), mid-turning vertical-angular speed peak (p < 0.05), mid-turning vertical-angular speed average (p < 0.05) and gait phases duration (p < 0.05) were significantly associated with age (Table 3). Stratification of the participants into subgroups according to 10 s iTUG time periods (Podsiadlo and Richardson 1991) or according to the cutoff value of 13.5 s (Shumway-Cook et al. 2000) showed some similarities. Generally, in both stratification cases significant differences between the subgroups of different mobility were found in vertical acceleration range, mid-turning variables and gait phases duration (Table 3).

**Table 3:** Mean (±SD) or median (IQR) values of the instrumented Timed-Up-and-Go test variables for total subjects number as well as for number of subjects stratified according to iTUG total performance time (r—correlation between age and a given variable for total subjects number)

Variable	Mean ± SD or median (IQR)						r
	Total subjects number (n = 26)	ITUG range: 10.01–20.00 s (n = 11)	ITUG range: 20.01–30.00 s (n = 10)	ITUG range: >30.01 s (n = 5)	ITUG range: <13.5 s (n = 3)	ITUG range: ≥13.5 s (n = 23)	
Sit-to-stand phase duration (s)	2.18 (0.97)	1.94 ±0.66	2.31 ±0.73	3.62 ±1.88	1.93 ±0.66	2.20 (1.21)	0.393 p < 0.05
Sit-to-stand anterior-posterior acceleration range (m/s^2^)	6.55 ±1.86	7.41 ±1.90	6.00 ±1.41	5.78 ±2.15	8.13 ±2.00	6.35 ±1.78	−0.254 n.s.
Sit-to-stand medial–lateral acceleration range (m/s^2^)	1.95 (1.20)	2.99 ±1.29	1.50 (0.90)	1.56 ±0.17	3.57 ±1.36	1.60 (1.25)	−0.327 n.s.
Sit-to-stand vertical acceleration range (m/s^2^)	4.21 ±1.63	5.28 ±1.44	3.60^a^ ±1.11	3.06^b^ ±1.69	6.53 ±1.00	3.90^*^ ±1.44	−0.578 p < 0.01
Mid-turning phase duration (s)	4.76 (4.16)	3.52 ±0.98	5.36 ±2.46	10.88^bbb^ ±3.66	2.74 ±0.41	5.42^*^ (3.95)	0.270 n.s.
Mid-turning vertical-angular speed peak (deg/s)	101.80 ±40.66	134.98 ±35.34	87.74^a^ ±22.13	56.94^bbb^ ±8.31	158.37 ±42.82	94.43^*^ ±34.91	−0.441 p < 0.05
Mid-turning vertical-angular speed average (deg/s)	36.20 ±17.06	50.96 ±12.19	30.50^a^ ±9.47	15.14^bbb^ ±3.29	63.93 ±5.87	32.59^**^ ±14.46	−0.418 p < 0.05
Gait phases duration (s)	8.64 (6.47)	5.58 ±1.79	10.16^aa^ ±3.14	17.71^bbb^ ±6.82	3.70 ±1.64	8.93^*^ (5.77)	0.450 p < 0.05
End-turning phase duration (s)	4.99 ±2.07	4.14 ±1.38	4.49 ±1.79	7.84^b,c^ ±1.47	3.87 ±0.85	5.13 ±2.15	0.130 n.s.
End-turning vertical-angular speed peak (deg/s)	92.87 ±27.31	105.28 ±31.67	91.82 ±16.28	67.68^b^ ±18.42	107.50 ± 47.19	90.97 ±24.75	−0.181 n.s.
End-turning vertical-angular speed average (deg/s)	34.13 ±12.89	39.36 ±13.05	36.35 ±9.49	18.20^bb,cc^ ± 2.32	38.10 ±5.86	33.62 ±13.54	−0.135 n.s.
End-turning stand to sit (s)	5.80 ±2.39	4.40 ±1.34	5.48 ±1.36	9.52^bb,c^ ±2.06	3.94 ±0.72	6.04 ±2.44	0.320 n.s.
Stand-to-sit phase duration (s)	2.75 ±0.98	2.25 ±0.80	3.12 ±0.99	3.11 ±1.02	2.37 ±0.38	2.80 ±1.03	0.255 n.s.
Stand-to-sit anterior-posterior acceleration range (m/s^2^)	7.30 ±1.76	7.74 ±1.87	7.19 ±1.44	6.54 ±2.18	7.33 ±1.39	7.29 ±1.83	−0.336 n.s.
Stand-to-sit medial–lateral acceleration range (m/s^2^)	4.38 ±1.36	5.04 ±0.76	3.97 ±1.25	3.74 ±2.13	4.97 ±1.31	4.30 ±1.38	−0.092 n.s.
Stand-to-sit vertical acceleration range (m/s^2^)	6.45 (7.28)	9.65 ±4.52	7.04 ±4.13	3.80 (4.00)	7.40 ±2.00	6.40 (8.10)	−0.255 n.s.

^a^ Comparison between iTUG variables obtained in subgroups 10 s < iTUG ≤ 20 s and 20 s < iTUG ≤ 30 s (^a^—p < 0.05; ^aa^—p < 0.01; ^aaa^—p < 0.001)

^b^ Comparison between iTUG variables obtained in subgroups 10 s < iTUG ≤ 20 s and iTUG > 30 s (^b^—p < 0.05; ^bb^—p < 0.01; ^bbb^—p < 0.001)

^c^ comparison between iTUG variables obtained in subgroups 20 s < iTUG ≤ 30 s and iTUG > 30 s (^c^—p < 0.05; ^cc^—p < 0.01; ^ccc^—p < 0.001)

^*^ Comparison between iTUG variables obtained in subgroups <13.5 s and **≥**13.5 s of total iTUG performance time (^*^—p < 0.05; ^**^—p < 0.01; ^***^—p < 0.001)

Multiple regression analysis for age as dependent variable revealed that the sit-to-stand vertical acceleration range was the only independent variable statistically significant (Table 4), which alone and independently from other variables explains 14.0% of the age variability (semipartial correlation = −0.374).

**Table 4:** Results of standard multiple regression analysis between dependent variable (age) and independent variables (sit-to-stand phase duration, sit-to-stand vertical acceleration range, mid-turning vertical angular speed peak and average, gait phases duration)

Dependent variable	r^2^	SEE	Independent variable	ß ± SE of ß	B ± SE of B	p
Age	0.430	**±**3.041	Intercept	–	82.439 ± 6.382	<0.001
			Sit-to-stand phase duration	0.307 ± 0.202	2.537 ± 1.667	n.s.
			Sit-to-stand vertical acceleration range	−0.544 ± 0.245	−1.204 ± 0.543	<0.05
			Mid-turning vertical-angular speed peak	0.165 ± 0.397	0.015 ± 0.035	n.s.
			Mid-turning vertical-angular speed average	0.137 ± 0.353	0.029 ± 0.075	n.s.
			Gait phases duration	0.278 ± 0.269	1.821 ± 1.766	n.s.

Subjects’ age significantly correlated with absolute (p < 0.05) and relative (per kg of FFM) values of IKEPT (p < 0.05) as well as with the results of 30 sCST (p < 0.05) and 6MWT (p < 0.05). The sit-to-stand vertical acceleration range (STSVAR) was not significantly associated with body weight, FFM and absolute or relative values of IKEPT (p > 0.05). However, significant correlations were noted with %Fat (p < 0.05) and the results of functional tests—30sCST (p < 0.01) and 6MWT (p < 0.01) (Table 5). Additionally, IKEPT did not correlated significantly with 30sCST (r = 0.276; p > 0.05), 6MWT (r = 0.116; p > 0.05), body mass (r = 0.219; p > 0.05) nor with FFM (r = 0.171; p > 0.05).

**Table 5:** Age and sit-to-stand vertical acceleration range associations and linear regression equations

Independent variable	Age		Sit-to-stand vertical acceleration range	
	r	Equation	r	Equation
Body weight (kg)	−0.129 n.s.	−0,038*x + 88.021	0.365 n.s.	0.048*x + 1.328
FFM (kg)	0.080 n.s.	0.048*x + 83.805	0.052 n.s.	0.014*x + 3.629
%Fat	−0.264 n.s.	−0.091*x + 88.518	0.415 p < 0.05	0.065*x + 2.253
IKEPT (Nm)	−0.392 p < 0.05	−1.950*lnx + 91.975	0.199 n.s.	0.446*lnx + 2.789
IKEPT/body weight (Nm/kg)	−0.361 n.s.	−1.854*lnx + 84.136	0.097 n.s.	0.224*lnx + 4.405
IKEPT/FFM (Nm/kg)	−0.407 p < 0.05	−2.061*lnx + 84.717	0.185 n.s.	0.422*lnx + 4.423
30-s CST (repetitions)	−0.422 p < 0.05	−0.560*x + 90.204	0.519 p < 0.01	0.310*x + 1.749
6MWT (m)	−0.482 p < 0.05	−0.025*x + 89.563	0.585 p < 0.01	0.014*x + 2.131

## Discussion

The objectives of this study were to determine the iTUG variable that is the best predictor of age, and to assess if this variable corresponds to the results of commonly used functional tests in elderly. The results showed that sit-to-stand vertical acceleration range (STSVAR) correlates the best with the subjects’ age and this variable is significantly associated with 30sCST and 6MWT results. Data stratification of the iTUG total performance time [based on that proposed by Podsiadlo and Richardson (1991)] revealed that none of the investigated women had normal mobility (iTUG score <10 s) and 57.7% of the subjects had impaired mobility (iTUG score >20 s). Our results are similar with those reported by other researchers regarding the nursing home women (Bischoff et al. 2003; Nordin et al. 2008). Additionally, considering the obtained in this study the 6MWT (total distance or average gait velocity) scores, and proposed in literature the cutoff values for TUG test for frailty (Savva et al. 2013) and for sarcopenia (Martinez et al. 2015), altogether imply that the investigated women were frail and sarcopenic (iTUG total performance time >11 s). Of note, although average gait velocity estimated on the base of the 6MWT is not a true indicator of slowness in frailty syndrome [according to frailty criteria given by Fried et al. (2001) the appropriate distance for gait velocity measurement is 4.572 m], the participants’ 6MWT score (distance) was substantially lower compared to the data reported by Steffen et al. (2002) for this age group.

The TUG is often used to identify the subjects at risk of falling (Shumway-Cook et al. 2000). Our data stratification by the cutoff value of 13.5 s (Shumway-Cook et al. 2000) showed that 88.5% of investigated women were in the risk of fall. Additional iTUG subtask variables analysis revealed the significant difference in STSVAR, gait phases duration and mid-turning variables between the subgroups of higher (>13.5 s of iTUG score) and lower (<13.5 s of iTUG score) fall risk. Although our results should be interpreted with a caution (because of the small sample size) they are supported by other studies findings indicating that muscle weakness, gait and balance disorders are important fall-risk factors (Rubenstein and Josephson 2002).

According to the phenotype model of frailty proposed by Fried et al. (2001) one of the frailty criteria is weakness or low muscle strength. Typically, this criterion is checked by handgrip maximal contraction since it has been shown that this muscle group is the most representative of the general strength of the muscles in the body (Rantanen et al. 1994). The other groups of muscles like quadriceps femoris are tested as well in older people, showing a good to moderate correlation with the handgrip strength (Lauretani et al. 2003; Peolsson et al. 2001). Taking into account that total TUG performance time can be used as a frailty discrimination factor, this implies that muscle strength should be an important factor in TUG performance. However, the results of the research dealing with the TUG time and muscle strength are equivocal. Some of them showed the significant correlation between the TUG time and a handgrip (Borges et al. 2015) or knee-extension strength (Kwan et al. 2011; Benavent-Caballer et al. 2016), while others did not observe such a relationship (Silanpää et al. 2014). These discrepancies could be at least partly clarified by the results obtained in this study namely a better association of the STSVAR than the IKEPT with iTUG total performance time.

It is well known that muscle strength declines with age, mainly because of the sarcopenia (Cruz-Jentoft et al. 2010). In consequence, muscle weakness leads to mobility restriction what is reflected in decreased functional test performances. In fact, a close relationship has been found between 6MWT (Bean et al. 2002b; Ozalevli et al. 2007) as well as 30-s CST (Hughes et al. 1996; Moxley Scarborough et al. 1999) performances and lower limb muscle strength. With regards to muscle strength, our results are in agreement with the above mentioned age-related association. Interestingly, the IKEPT was not significantly correlated with the results of 30-s CST and 6MWT, although the functional test performances were dependent on participants age and significantly correlated with iTUG total time. Because of the limited data available, it is difficult to explain these findings. However, considering the facts that: (a) a sample of investigated women was of advanced age with impaired functional ability (see the results of iTUG or 6MWT), (b) there is a lack of significant association between IKEPT and body weight or FFM, (c) STSVAR is positively correlated with percentage of body fat, and (d) knee extensors, along with hip extensors, are an important muscle group involved in walking as well as in rising from the chair (Bean et al. 2002b; Hughes et al. 1996), altogether may indicate the lower muscle quality i.e. the ability to develop strength per unit of muscle mass (Morse et al. 2005) and/or a change of muscle properties i.e. a decline in muscle power. Indeed, it has been shown that lower limb muscle power has a greater impact on the functional test performance than muscle strength alone in community-dwelling older people (Bean et al. 2002a) as well as in residents of chronic care hospital (Bassey et al. 1992).

An additional confirmation of above mentioned explanations for failing to find any association between IKEPT and functional test performances may be the identification of STSVAR as the best iTUG predictor of the women’s age. It is well recognized that sit-to stand (STS) movement can be more biomechanically demanding than other activities like walking or stair climbing. Since the movement from a stable to a less stable position takes place, it requires greater muscular efforts and greater joint ranges of motion (Hughes and Schenkman 1996). There are two main strategies for rising from the chair. In the first one, which called the momentum transfer strategy, subjects use momentum generated by the trunk to aid in rising. In the second one, called stabilization strategy, very little momentum is generated and movements which increase stability are employed (Hughes and Schenkman 1996). It has been reported, that during STS movement the older people tend to flex the trunk more, increase their hip flexion velocity and gain a higher momentum than young subjects. This increase in momentum helps overcome the increased torque required to be generated by the knee extensors when rising from the chair. In consequence, global muscular effort required to move the body forward and gain stability is lowered and lasts a shorter time; nevertheless, the maximal speed of execution of this motor task is significantly lower compared to young individuals (Papa and Cappozzo 2000). The described above mechanism demonstrates that elders through this strategy minimalize the usage of knee extensors in the STS task. In fact, a significant correlation between %Fat and STSVAR found in this study may corroborate this chair rise strategy since aging is associated with increased accumulation of visceral fat (Hunter et al. 2010) what in consequence may gain a higher momentum during trunk flexion. On the other hand, Roebroeck et al. (1994) found that although forward rotation of the trunk contributes to the velocity of the body’s center of mass in horizontal direction, the extension of the lower limbs contributes this velocity in vertical direction. Given that any changes in velocity of the body’s center of mass occurring during a very short period of time results in a large acceleration (Lowry et al. 2013), it suggests that the rate of muscle force development may be of relevance to STS task execution. Furthermore, the findings of this study, namely identifying STSVAR as the best iTUG predictor of the participants’ age, and the lack of correlation between IKEPT and test performances, imply that in older persons the functional mobility is more related to the explosive force of lower limb extensors than to theirs maximal isometric strength. This suggestion appears to be a relevant factor also in walking tasks as well since STSVAR was significantly positively correlated with a walking distance in the 6MWT what is in line with the findings reported by Bean et al. (2002b).

The findings of this study may have some practical implications for nursing home staff (nurses, physiotherapists and other health providers). Given that muscle strength decreases with age, what can affect older adults daily life activities (e.g., getting out of a chair or bath tub or getting out of a bed), elderly persons with low knee extensors strength should be taught a proper technique how to rise from a chair (bending the trunk during chair rise to increase the momentum). This will allow nurses to make less efforts to help elderly persons in sit-to-stand transitions. Moreover, considering that with advancing age muscle power declines earlier (Reid and Fielding 2012) and its impairment is more influential determinant of mobility performance than impairment in muscle strength, the rehabilitation programs in elderly adults should include the interventions enhancing muscle power and emphasizing high muscle contraction velocity.

There are several limitations to this study. Firstly, the sample of our study participants was restricted to institutionalized oldest old women and, therefore, may not be representative of general elderly population at this age. Secondly, the cohort of institutionalized women was relatively well functioning. Thus, the results of this study are applied to individuals who could perform the tests. Thirdly, beside the analyzed iTUG we used in this study only two additional functional tests (30sCST and 6MWT). We included these tests in order to assess lower limb strength and functional exercise capacity. However, the measured variables may not cover all physical information that explains the variability in the iTUG. Finally, the generalization of the findings in this study is limited by the small sample size; therefore, these results should be interpreted with caution and considered as preliminary research.

In summary, iTUG testing performed on the sample of nursing home women identifies STSVAR as the best predictor of participants’ age. The close relationships between STSVAR and 30-s CST along with 6MWT, but insignificant correlation with IKEPT, suggest that functional test performance is more dependent on the rate of force development than on maximal isometric strength of lower limb muscles. These findings may partly clarify the diversity of strength measurement outcomes in elderly populations reported in the literature. Moreover, these findings establish the link among age, TUG performance subtasks, and lower limb muscle strength in older adults. Future studies are needed to determine the generalizability of these results to larger cohorts of institutionalized and non-institutionalized elders that differ in health (frailty) status.
